# Integrating climate and health: A national survey of medical societies' actions and barriers

**DOI:** 10.1016/j.joclim.2026.100665

**Published:** 2026-03-23

**Authors:** Tracey L. Henry, Sonya Vijayvargiya, Onyie Eze, Avni Ahuja, Mehul Tejani, Tola Ebunlomo, Olivia Cote

**Affiliations:** a Associate Professor, Emory University School of Medicine, Division of General Internal Medicine, USA; b Medicine Resident Intern, Weill Cornell University School of Medicine, NY, NY, USA; c OB-GYN Resident Intern, University of Pennsylvania, Philadelphia, PA, USA; d OBGYN Resident, USA; e Medical Student, Emory University School of Medicine, Atlanta, GA, USA; f Opthalmology Resident, University of Colorado Anschutz Medical Campus, Aurora, CO, USA

**Keywords:** Climate change, Health, Policymaking, Strategies, Advocacy, Climate justice

## Abstract

**Introduction:**

A growing number of clinicians and clinical leaders are acknowledging the impacts of rising carbon emissions and committing their organizations to decreasing their carbon footprint. In 2016, the Medical Society Consortium on Climate and Health (MSCCH) leveraged its national power and rallied major medical societies to take a stance on climate-related issues. This study assesses the extent to which medical societies within MSCCH are addressing climate change through emissions reduction efforts, research, education and policy and other programming required to reduce their carbon footprint.

**Materials:**

A ten-question survey study was reviewed and approved by the MSCCH in 2022. The survey was disseminated to all thirty of the MSCCH’s member societies between 2022 and 2023. Twenty-two member societies responded. We measured the proportion of societies implementing initiatives to reduce their carbon footprint and improve education/advocacy on climate topics and summarized self-reported society-level barriers in achieving reductions in carbon emissions.

**Results:**

Sixteen societies (73%) enacted measures to reduce carbon emissions. Four societies (18%) pledged to reduce their carbon emissions to varying degrees; only two had a strategic plan for achieving these goals (1%). Nineteen societies (86%) implemented measures to improve education and advocacy around climate change. The most common barriers to climate-conscious advocacy were a lack of staff time and lack of funding.

**Conclusions:**

This work highlights the pivotal role medical societies play in operationalizing the integration of climate and health policy. Policymakers and other relevant parities must promote investment and regulatory action, education and research supporting decarbonization.

## 1 Introduction

The effects of climate change on human health are diverse and affect every medical specialty and stage of life from birth to geriatric or end of life care [1]. From respiratory disease exacerbations from wildfires to heat-related illnesses, climate change has been associated with increasing numbers of premature death [2]. Between 2004 and 2018, an average of 703 heat-related deaths occurred annually in the United States [3].

The healthcare sector is one of the largest contributors to global carbon emissions. These emissions have not been justified by a commensurate improvement in health status or benefits. Globally, the healthcare industry is responsible for nearly 4.5 % of all emissions and toxic air pollutants [4]. The US, which accounts for the largest share of global healthcare emissions, estimates that 8–10 % of all domestic emissions are from healthcare-related industries [5]. The health effects of pollution attributable to the healthcare industry are estimated to be similar to the number of deaths due to avoidable medical errors [6].

Clinician leaders, including hospitalists, are beginning to acknowledge the impacts of rising carbon emissions and are committing to decreasing their carbon footprint although unclear of their pathway to engagement [7]. The Federal Government passed the Inflation Reduction Act in 2022 which aims to reduce the effects of climate change by promoting clean, renewable energy [8]. Additionally, over 100 health sector leaders, health systems, hospitals, industry organizations and nonprofits previously signed the White House Pledge to reduce greenhouse gas emissions by 50 % by 2030 and to reach net-zero emissions by 2050 [9]. The National Academy of Medicine launched the Action Collaborative on Decarbonizing the U.S. Health Sector, a network of leaders committed to addressing the health care sector’s environmental impact while strengthening its sustainability and resilience [10].

Medical societies are major agents of change in the healthcare sector and help drive policy, advocacy, research and standards that impact both patients and the healthcare profession. Thus, in 2016, the Medical Society Consortium on Climate and Health (MSCCH) rallied major medical societies, now representing over 1 million physicians and health professionals, to tackle climate health-related issues. Here we summarized the actions taken by MSCCH societies and identified gaps in resources and initiatives in reducing their carbon footprint.

To accomplish goal, we surveyed medical societies within the MSCCH to assess which steps medical societies are taking to mitigate their carbon footprint and to promote climate health education and advocacy within their organizations.

### 1.1 Methods

The authors created a 10-question survey to evaluate the actions the Medical Society Consortium on Climate and Health (MSCCH) societies are taking to reduce their carbon footprint and promote awareness about climate change. Survey questions were developed by the study authors based on the MSCCH Consensus Statement and its climate action priorities. Draft items underwent content review by MSCCH leadership for clarity, relevance and alignment with Consortium goals. The instrument was not externally validated, as the study was designed as an exploratory descriptive assessment. The rationale for the survey questions was informed by the MSCCH’s consensus statement which underscores the responsibility of health professionals and their medical societies to adopt sustainable practices to reduce their carbon footprints via awareness of actual contribution of their individual and collective carbon emissions, educating the public and policymakers, taking other preventive and protective measures that contribute to sustainability, clinicians and hospitals adopting sustainable practices, and implementing comprehensive and economically sensitive approaches to limiting climate change [11]. The survey queried medical societies concerning gaps in resources, education, and other programming required to reduce their carbon footprint as prioritized by the MSCCH’s consensus statement that highlights and supports the 2016 U.S. Climate and Health Assessment – The Impacts of Climate Change on Human Health in the United States: A Scientific Assessment. The survey was reviewed and approved by the MSCCH in 2022 and disseminated via email to all of its then total of 30 MSCCH’s member societies in 2022 and 2023, representing over 500,000 clinicians (supplement 1). The MSCCH consists of both member societies and affiliate organizations. Only the 30 MSCCH member societies that were in existence in 2022 and 2023 and represented national medical societies were included in this study. Affiliate organizations (>100) were not surveyed because they differed structurally and included health-related organizations, advocacy groups, or smaller professional groups. Responses were summarized using Microsoft Excel. This study received an Institutional Review Board exemption from Emory University School of Medicine.

#### 1.1.1 Study participants

**Fig. 1:**
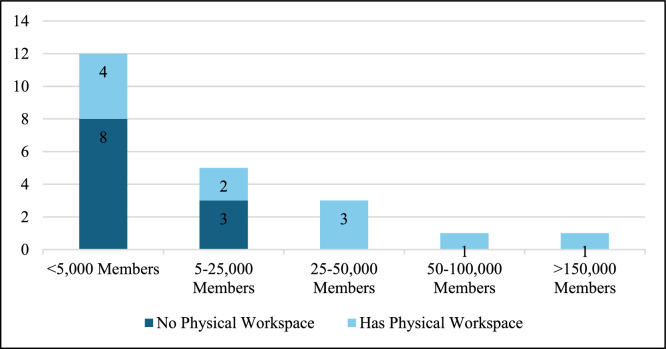
Society demographics.

All 30 of the MSCCH’s member societies that were members in 2022 and 2023 were invited to participate. Twenty-two member societies participated. See Fig. 1 for member society demographics.

### 1.2 Results

There were 26 total responses, as four societies took the survey in both 2022 and 2023. We excluded the first submission for participants who completed the survey twice (*n* = 4) to ensure reporting of the most recent information without redundancy. Of the 22 remaining submissions, 20 were complete survey submissions. 12 participating societies had <5000 members (55 %), 5 had 5–25,000 members (23 %), 3 had 25–50,000 members (14 %), 1 had 50–100,000 members (5 %), and 1 had >150,000 members (5 %).

The survey revealed that 4 societies assess their carbon footprint (18 %). Of current measures being taken by societies to reduce carbon footprint, 11 (50 %) reported having a dedicated physical office or headquarters from which staff operate, while the remaining societies functioned primarily virtually or through distributed administrative support without a central physical office; 5 (23 %) encouraged recycling; 4 (18 %) enacted timed lighting systems; and 1 (4.5 %) had done so after switching to LED lighting. Other efforts reported by participants included installing efficient hot water and cooling systems, encouraging public transportation through assistance programs, reducing transportation by increasing the number of virtual meetings through telework, educating members with electronic materials, minimizing plastic use at meetings and conferences, and maintaining the physical office space as Leadership in Energy and Environmental Design (LEED) Gold certified. Fig. 1 illustrates both the number of societies that are currently enacting specific measures and highlighting the actual initiatives to reduce their carbon emissions.

Furthermore, 12 societies passed resolutions or internal policies on climate change. Nine societies (75 %) passed one or two resolutions, 2 societies passed 3 or 4 resolutions (17 %), and one society (8 %) passed 5 or more resolutions.

Four societies pledged to reduce their carbon emissions. Reduction targets included carbon neutrality by 2030 (*n* = 1), a 50 % reduction by 2030 (*n* = 1), making the society’s annual meeting carbon neutral (*n* = 1), and enacting virtual newsletters and meetings (*n* = 1). However, only 2 societies had a strategic plan for achieving these goals. Further, as shown in Fig. 3, 8 societies (36 %) reported dedicating staff time to climate related work, and 2 societies (41 %) reported having an established climate or environmental sustainability task force.

**Fig. 2:**
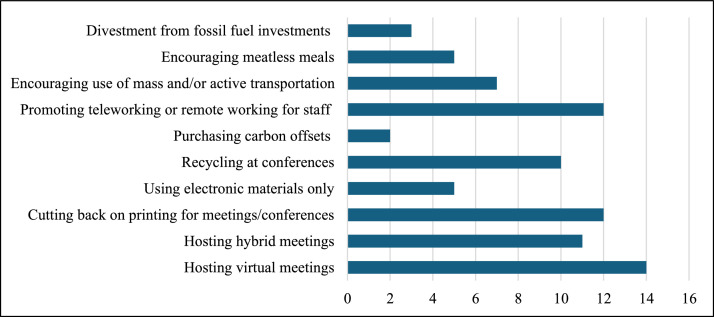
Number of medical societies currently enacting measures to reduce carbon emissions.

In addition to current measures to reduce their carbon footprint, some societies had previously enacted measures to improve education and advocacy regarding the climate crisis (Fig. 2). The most common measures implemented were providing continuing medical education (CME) or education around climate change (84 %) and conducting or publishing research on climate change (68 %).

**Fig. 3:**
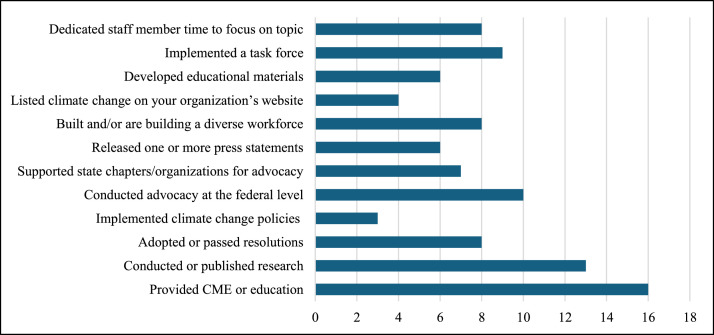
Number of societies that have implemented measures to improve education and advocacy.

Among the 4 societies that took the survey in both 2022 and 2023 (Society of Reproductive Endocrinology and Infertility, American Psychiatric Association, American College of Physicians, and Society for Pediatric Dermatology), there were no changes in the presence or absence of physical working space, assessment of the society’s carbon footprint, pledge to reduce carbon emissions, strategic plan to reduce carbon emissions, or the number of resolutions passed related to climate change.

In the period between the initial and subsequent survey completion, 2 of the 4 societies did not enact any new measures to offset carbon emissions. However, one society officially began reducing the number of printed materials and implementing recycling at conferences, while another officially began divesting from its fossil fuel investments.

Two societies did enact new measures in the inter-survey period. One supported state chapters/organizations advocating for climate change on a state or local level and developed educational materials for their organization members/relevant patient population. Another society indicated that it conducted advocacy on climate change and its effect on health at the federal level. Both societies reported that they were building a diverse workforce to address climate and health equity issues. Notably only one medical society listed an estimate for their carbon footprint, which begs the question of how are medical societies truly capturing the effects of their environmental sustainability efforts if unclear of their contribution to US carbon emissions.

**Fig. 4:**
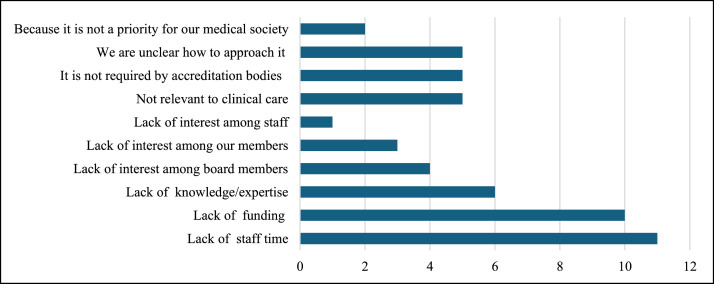
Barriers to medical society climate change advocacy.

Within the larger society sample, several barriers to enacting further changes were identified (Fig. 4), with the most common being a lack of staff time (57 %), followed by a lack of funding (53 %), and a lack of knowledge/expertise (32 %). Other barriers to addressing climate change include a lack of buy-in from society membership and difficulties linking climate change directly to members' clinical practice, which is typically the primary focus of their membership. Societies noted that they are also inclined to focus on the clinical ramifications of climate change rather than politics, given the presence of members who are climate change “deniers” or who deem the topic to be “too political.”

### 1.3 Discussion

This survey highlights the different approaches medical societies have adopted to address climate change. Many of these approaches center on educating their membership about climate change, educating patients, research endeavors and policy approaches. Within societies that had physical spaces to work in, members encouraged recycling initiatives, energy conservation, and alternative modes of transportation. Four societies pledged to reduce their carbon emissions with goals spanning carbon neutrality by 2030, a 50 % reduction in carbon emissions by 2030, making the society’s annual meeting carbon neutral, and implementing virtual newsletters and meetings. However, only 2 of these societies had a strategic plan or framework for achieving these goals.

Survey responses also reveal some of the systemic challenges societies encounter when attempting to enact environmentally conscious interventions. These include a lack of funding, staff resources, and member interest. Other studies have also identified that clinicians experience several barriers that prevent them from incorporating information about climate change into their practice or other professional activities. Survey respondents from member societies highlighted the persistent need for climate-related professional education, policy statements on climate change, and training regarding sustainability practices in the workplace. Further, drivers of health professionals’ willingness to engage in climate advocacy included a higher rate of perceived professional consensus around health harms from climate change [5]. In other words, “an influential set of trusted voices” may prompt more clinicians to act against climate change and its health effects [12].

It is worth noting that the power to enact climate-friendly interventions is not equal among society members. Over half of the survey respondents represented societies with <5000 members. Smaller societies may not have the financial resources to create patient and member materials or allocate funding to climate change research. Some societies own their buildings while others rent, which may determine how much autonomy they have in establishing climate-conscious infrastructure. In this regard, we believe larger medical societies should take a proportional role in setting practice guidelines to address the climate crisis. We encourage societies to improve transparency with their membership, specifically relating to the implementation of climate-conscious interventions. By doing so, societies demonstrate actionable improvements for providers and payers to follow thereby potentially motivating their membership to engage more directly with climate change advocacy and policy.

Further, education, research and advocacy are urgently needed to support climate action to mitigate the deleterious effects of our current climate crisis. The harms arising from increasing CO_2_ emissions and global temperatures must be acknowledged by the healthcare community. Medical societies are particularly poised to promote environmental sustainability given their innate roles in the healthcare sector as drivers of healthcare policy and advocacy, research agendas and professional education and guidelines. Investment in regulatory action supporting decarbonization are key ways that healthcare organizations can specifically drive progress in reducing healthcare carbon emissions. Several partnerships already exist. Organizations such as Health Care Without Harm, Practice Greenhealth, My Green Doctor and Energy Star have put forth detailed recommendations for minimizing the carbon footprint of the industry and improving building efficiency. Each specialty and its corresponding society should task themselves with understanding both how they explicitly contribute to carbon emissions and how rising emissions affect their specific patient population. Given climate change is a threat multiplier to marginalized communities, having a workflow in place to assess and address a patient’s climate risk via climate health history can lead to climate-informed healthcare and delivery and improved health outcomes [5], [13].

#### 1.3.1 Education

Health Professional education is also a necessary element for promoting progress in this area. Medical students along with health professionals also feel unprepared to engage in climate interventions and advocacy at the patient, community and legislative levels [5]. Longitudinal climate health curricula for all health professionals across undergraduate medical education (UME), graduate medical education (GME) and continuing medical education (CME) are needed to ensure competency in clinical practice and healthcare sector advocacy and policy engagement. A top-down approach to setting educational priorities, e.g. in the form of CME, can influence accrediting organizations to incorporate certification requirements that address topics related to climate change, similarly an approach for accrediting bodies for UME and GME. Moreover, offering CME for climate-related courses could increase voluntary participation. Climate and health competencies are increasingly recognized by certifying bodies. Notably, the American Board of Internal Medicine has now incorporated climate related questions into its certification blueprint and the American Board of Pediatrics offers climate and health educational content that is eligible for Continuing Medical Education (CME) and also fulfills Maintenance of Certification requirements [14,15]. Climate integrated education and reform are pivotal to addressing our climate crisis and contributing to a more sustainable healthier planet [16].

#### 1.3.2 Research

Climate change and environmental science research to advance the health and wellness of our patients, communities and the planet at large is key to developing clean energy solutions for more climate resilient communities and better health outcomes. Scientific research drives innovation to meet climate goals via more green technologies such as renewable energy, energy efficient solutions and sustainable transportation. Translational, clinical and basic science and epidemiological research determines both the current gaps and effectiveness of current policy, interventions, metrics on mitigating the effects of climate change. We must continue to invest in a robust research agenda and funding to move the needle toward more sustainability in healthcare. Gaps in research and innovation must center on the gaps in advancing the linkage between climate change, health equity and community partnership [17].

#### 1.3.3 Health policy and advocacy

Not all MSCCH member societies have position statements or information for their members to implement policies, initiatives or guidelines in their clinical practice. Medical societies should take on a more active role in providing guidance to their members and set practice guidelines to confront the climate crisis [18]. For the dual purpose of reining in long-term healthcare costs and simultaneously improving population health outcomes, policy statements on climate change should not just highlight the exacerbating effect of the healthcare sector on climate change but actionable strategies. These statements serve also as policy recommendations for governmental and regulatory agencies to support sustainability in healthcare delivery. It is important to encourage, if not mandate, the decarbonization of the industry and advocate for broad engagement across relevant parties to lower overall emissions and improve the health of our nation and our planet.

### 1.4 Conclusions and call to action

Based on the gaps identified in our survey, including lack of staff capacity, limited funding, and inconsistent strategic planning, medical societies must mobilize to actively support and promote scientific integrity. This can be achieved by urging Congress and federal agencies to inform their regulatory actions with the use of peer-reviewed research and sound methodology. During precarious political times, as part of our chosen profession, medical professionals and by proxy their medical societies have a duty to advocate for evidence-based policies that protect the health of our nation. We should use our voices to educate policymakers and the public not only about how climate change harms our health and wellbeing but how reversing federal emissions regulations can undermine our health and disease prevention. Finally, we have to continue to amplify the voices of the marginalized, recognizing that the communities most affected by the climate crisis have contributed least to the problem but bear most of the burden. As health professionals we have a moral imperative to defend health, science and our shared future.

## CRediT authorship contribution statement

**Tracey L. Henry:** Writing – review & editing, Writing – original draft, Supervision, Resources, Project administration, Methodology, Formal analysis, Data curation, Conceptualization. **Sonya Vijayvargiya:** Writing – review & editing, Methodology, Formal analysis. **Onyie Eze:** Writing – review & editing, Writing – original draft, Methodology, Formal analysis, Data curation. **Avni Ahuja:** Writing – review & editing, Writing – original draft. **Mehul Tejani:** Writing – review & editing. **Tola Ebunlomo:** Writing – review & editing. **Olivia Cote:** Writing – review & editing, Writing – original draft, Methodology, Formal analysis.

## Declaration of competing interest

The authors declare that they have no known competing financial interests or personal relationships that could have appeared to influence the work reported in this paper.

## Appendix Supplementary materials

Image, application 1
