# Supplementary material for: Integrating climate and health: A national survey of medical societies' actions and barriers
Source: J Clim Chang Health. 2026 Mar 23;28:100665. doi: 10.1016/j.joclim.2026.100665 (PMC13049539; doi:10.1016/j.joclim.2026.100665)
Supplement: Supplementary file 1 [file mmc1.pdf]

Survey to Assess the Climate Actions of Medical Societies

Purpose

The Medical Society Consortium on Climate and Health in partnership with National Medical Association, is conducting this survey to assess what actions and/or initiatives medical societies are taking to mitigate their carbon footprint and support climate education and health for their members and relevant patient populations.

As the health sector is one of the largest contributors of carbon emissions, data from this survey will be used to inform policy makers on what is being done by medical societies to meet federal goals to reduce carbon emissions by 50 percent by 2030 and net zero by 2050. It will also provide a gap analysis of resources, educational needs and other programming required by medical societies to reduce their carbon footprint.

Participation

This survey is voluntary. You may withdraw from the survey at any time and for any reason.

You must be 18 years of age or older in order to participate in this study.

Confidentiality

All surveys will be confidential. Names and other identifiers will not be placed on surveys or other research data.

This survey will take approximately **10 minutes** to complete and will be open until October 31, 2022 at 5PM EST.

Consent

I have read this form, am at least 18 years of age, and agree to participate in this study.

I do not agree to participate in this study and/or I am under the age of 18.

Demographics

Name of medical society (include a drop box of choices of member societies)

- Academic Pediatric Association (APA)
- Academy of Integrative Health and Medicine (AIHM)
- American Academy of Allergy Asthma & Immunology (AAAAI)
- American Academy of Dermatology (AAD)
- American Academy of Family Physicians (AAFP)
- American Academy of Ophthalmology (AAO)
- American Academy of Pediatrics (AAP)
- American Academy of Physical Medicine and Rehabilitation (AAPM&R)

- American Association for Community Psychiatry (AACP)
- American College of Emergency Physicians (ACEP)
- American College of Emergency Physicians, California (CalACEP)
- American College of Emergency Physicians, Wisconsin (WACEP)
- American College of Lifestyle Medicine (ACLM)
- American College of Obstetricians and Gynecologists (ACOG)
- American College of Occupational and Environmental Medicine (ACOEM)
- American College of Osteopathic Internists (ACOI)
- American College of Physicians (ACP)
- American College of Preventive Medicine (ACPM)
- American College of Radiology (ACR)
- American Geriatrics Society (AGS)
- American Medical Association (AMA)
- American Medical Women's Association (AMWA)
- American Podiatric Medical Association (APMA)
- American Psychiatric Association (APA)
- American Society for Reproductive Medicine (ASRM)
- American Society of Cataract & Refractive Surgery (ASCRS)
- American Society of Clinical Oncology (ASCO)
- American Society of Pediatric Neurosurgeons (ASPN)
- American Telemedicine Association (ATA)
- Association of Academic Physiatrists (AAP)
- College of Urgent Care Medicine (COUCM)
- Endocrine Society
- Infectious Diseases Society of America (IDSA)
- Medical Dermatology Society (MDS)
- National Hispanic Medical Association (NHMA)
- National Medical Association (NMA)
- Society for Academic Emergency Medicine (SAEM)
- Society for Pediatric Dermatology (SPD)
- Society for Disaster Medicine and Public Health (SDMPH)
- Society of Behavioral Medicine (SBM)
- Society of General Internal Medicine (SGIM)
- Society of Gynecologic Oncology (SGO)
- Wilderness Medical Society (WMS)

Not listed please write in ____________

Name of person completing survey

____

Email of person completing survey

____

What is your position in the medical society?
____

Size of medical society

- <5,000
- 5-25,000
- 25-50,000
- 50,000-100,000
- 100,000-150,000
- >150,000

Questions

1. Does your medical society have a physical space?
    - Yes
    - No

2. If your medical society has a physical space, what actions, if any, is it taking to be more sustainable (e.g. recycling, weatherization of windows, timed lighting, encouraging carpooling or public transport, minimizing single use plates/napkins/utensils)?

    Please list your top three initiatives______________
3. Does your medical society assess its carbon footprint?
    - Yes
    - No
    - Don't know

4. What is your medical society's operations carbon footprint (e.g. your national headquarters or office space and your national conferences or national meetings)?
    - Insert amount here ___________CO_2e/yr metric ton
    - Don't know
    - Comments (optional)

5. Has your medical society made a pledge to reduce its carbon emissions?
    - Yes
    - No

- Don’t know

  If yes, please describe the exact nature of the pledge (e.g. name of the pledge or describe the call to action of the pledge such as Biden’s administration call to reduce carbon emissions 50% by 2030 and net zero by 2050)_____________________

  What year did your medical society make the pledge? _________

6. Does your medical society have a strategic plan or framework to reduce its carbon emissions?
   - Yes
   - No
   - Don’t know

7. What is your medical society currently doing to reduce its carbon emissions? (Please check all that apply)
   - Hosting virtual meetings
   - Hosting hybrid meetings
   - Cutting back on printing for meetings/conferences
   - Using electronic materials only
   - Recycling at conferences
   - Purchasing clean energy
   - Purchasing carbon offsets
   - Promoting teleworking or remote working for staff
   - Encouraging use of mass and/or active transportation for staff and members
   - Encouraging meatless meals
   - Divestment from fossil fuel investments
   - Please write-in additional efforts ____________

8. Has your medical society implemented or adopted any of the following? (Please check all that apply)
      - American Medical Association (AMA): Declaring Climate Change a Public Health Crisis
      - National Medical Association (NMA): Climate Change, Health and Equity
      - American Academy of Pediatrics (AAP): Global Climate Change and Children’s Health: Policy Statement
   - Provided CME or education around climate change
   - Conducted research on climate change
   - Implemented climate change policies
   - Adopted or passed resolutions on climate change
      i. If so, how many resolutions have you passed on climate change?
         - 0

        - 1-2
        - 3-4
        - 5 or more
    - Conducted advocacy on climate change and its effect on health at the federal level?
    - Supported state chapters/organizations for advocacy on climate change on a state or local level?
    - Listed climate change as an organizational priority on your organization's website
    - Released one or more press statements about your climate change efforts
    - Developed educational materials for your organization members or relevant patient population
    - Built and/or are building a diverse workforce to address climate and health equity issues
    - Implemented a task force, working group or committee on climate change?
    - Dedicated staff member time to focus on climate change and/or environment

9. What barriers has your medical society experienced, if any, with regard to conducting climate change education and/or advocacy? (Please check all that apply)
    - Lack of knowledge/expertise
    - Our society lacks a climate change policy or resolution
    - Lack of staff time
    - Lack of interest among our members
    - Lack of interest among staff
    - Lack of interest among board members
    - Not relevant to clinical care
    - Lack of funding
    - We are unclear how to approach it
    - It is not required by accreditation bodies
    - None of the above, because it is not a priority for our medical society

10. Is there anything else you feel we should know to understand your medical society's views and/or actions with regard to climate change?
    ____
